# Why Porcelain Bridges Fail—The Remedy

**Published:** 1904-05-15

**Authors:** George W. Schwartz

**Affiliations:** Chicago, Ills.


### WHY PORCELAIN BRIDGES FAIL—THE REMEDY.

BY GEORGE W. SCHWARTZ, M.D., D.D.S., CHICAGO, ILLS.

It is my belief that the most prominent causes of failure with porcelain bridges in their order are:

A lack of knowledge as to how to work porcelain.

Not studying conditions.

Making porcelain bridges that are too long to stand the stress put upon them when instead a removable piece is indicated and should be made.

Not dividing the strength equally between the roots of the teeth, the metal work and the porcelain.

Not protecting the inter-proximal space at each abutment.

Making saddle bridges, setting them in cement, leaving excess cement under the saddle.

Lastly, but not least, lack of artistic restoration in replacing lost teeth and tissue.

For a dentist to try and make a porcelain bridge at this age of porcelain work without first acquiring a working knowledge of porcelain is an injustice to both his patient and himself. For him to have a smattering of porcelain ideas and then expect to deliver to his patient serviceable porcelain work in bridging is a mistake and an expensive experiment. There is no place in practical dentistry where he will be required to stand so much grief as failure in porcelain bridge-work. To avoid this he should first find where he can obtain the desired knowledge, then go to this source and get it. The money thus spent will be a small item compared to what his failures will amount to if he does not do so. He must learn how to decide on a bridge. He learn how to trim roots for a porcelain bridge. How to plan the bridge. How to make the metal-work. How to carve and bake. Also how to set a bridge. To get these cardinal points drilled into him it will be necessary for him to change some of his other prosthetic methods. He must be careful, patient, clean, methodical and gentle. He must bend to the courage of his convictions and follow them. He must demand of his patients the amount due him for this service so that he may feel justified in making a piece of work that will not be a failure and avoid unconsciously slighting his work.

There is no part of porcelain dentistry where "a little learning is a dangerous thing" more than in porcelain bridge-work.

We are now to the point of studying conditions. This can not be done unless the dentist has a thorough working knowledge of porcelain. He must take into consideration the age of the patient, whether a male or a female, the amount of stress that will come upon the bridge and in what direction, whether the abutments are exceptionally firm in their sockets or not, the amount of space the bite allows for porcelain, how long the span is to be and if it will stand the strain to be put upon it. This brings us to the next reason why porcelain bridges fail—that of making fixed bridges which are too long to stand the masticating force brought to bear upon them.

For years I have taught and written that many cases are bridged in porcelain and bridged with gold, where two teeth should have been crowned and a removable piece of work made for the case to get the best results, and it will take a stronger argument than I have yet heard to change my belief. Fixed porcelain bridges that are made where the span is too long to justify them, I count among the commonest of errors in porcelain bridge-work.

I would like every dentist who does porcelain work to commit this rule to memory: *Divide the strength equally between the roots of the teeth, the metal-work and the porcelain.*

Understand when I say divide the strength equally, I mean you should do so as nearly as is possible. One of the most difficult things I have had to do in teaching students was to impress on their minds the importance of cutting the roots down enough. In most cases they have been afraid to shorten them sufficiently. Bands for porcelain bridges do not need to be very wide. Dentists do not give enough attention to the detail of constructing the metal-work. The bands should be made of platinum, as it is more accurately and easily fitted to the roots. Iridio-platinum should be used for copes on the bands. Square iridio-platinum wire should be used in the caps for posts in the roots used for the abutments. Square posts enable us to remove the caps from the plaster models and replace them in position with more certainty than round posts do. But round iridio-platinum wire should be used for bars between the abutments, thus avoiding sharp angles to fracture porcelain in mastication. All joints should be soldered with twenty-five percent platinum solder. You can then be sure your work will go through the furnace unchanged and not come unsoldered at the joints. Platinum backings should be soldered on the lingual surface of the metal-work to support the added porcelain. By following this method I believe you will be pursuing the correct one to divide the strength as equally as possible between the abutments, the metal-work and the porcelain. In constructing the metal-work for a porcelain bridge the dentist fails who does not take into consideration the inter-proximal space at each abutment. The work at these points should be made so it will not encroach on the tissues. Neither should they be so shaped that they will retain food there. One of the serious objections in making saddle bridges and setting them with cement is the interproximal space is often obliterated and the tissues abused by the saddle bearing too hard at these places, together with not removing all excess cement from beneath the saddle when the bridge is set. It is imperative for dentists who make saddle bridges to learn how to set them with gutta-percha. If a porcelain bridge has been made and is a serviceable piece of work, but lacks artistic results, it still has an element of failure about it and by many patients would be considered a faulty piece of work. For the remedy we must again turn to the preparation of the roots of the teeth for abutments. The roots should be so prepared by beveling the labial or buccal margins that when the caps are made they can be covered by live looking porcelain. To do this the facings should be ground to cover the bands. You can not get the artistic results by baking porcelain body on the bands that you can by grinding the facings to cover them.—*Review.*

